# 4071 Dynamic Control of Tumor Vessels Augments Antitumor Responses

**DOI:** 10.1017/cts.2020.64

**Published:** 2020-07-29

**Authors:** Emmanuel M Gabriel, Deborah Bahr, Sanjay Bagaria, Debrabata Muhkopadhyay, Keith Knutson

**Affiliations:** 1 Mayo Clinic; 2 Mayo Clinic Florida

## Abstract

OBJECTIVES/GOALS: Our overall objective is to develop a directly observable and reproducible method of enhanced blood flow through tumor vessels (i.e. dynamic control) at the time of systemic treatment delivery. Our central hypothesis is that the dynamic control of tumor vessels will improve (1) systemic drug delivery and (2) effector cell trafficking to target tumor. METHODS/STUDY POPULATION: B16 melanoma cells were inoculated into C57BL/6 (B6) mice (male and female) in both regional (hind leg) and systemic (flank) models. Dynamic control consisted of an IV saline bolus (500 ul) and phenylephrine (10 ug). Tumor vessel response was observed in real-time through window chambers using intravital microscopy (IVM). Dynamic control was combined with melphalan (20 mg/ml) either regionally (isolated limb perfusion) or systemically. Outcomes included tumor growth, survival, IHC, and toxicity. Dynamic control will be combined with adoptive transfer of effector T cells. B6 mice will be inoculated with B16/OVA (flank with window chamber) and treated with fluorescently labeled (calcein), OVA-specific CD8+ T cells from OT-1 transgenic mice. IVM, IHC, and flow cytometry will be used to measure T cell trafficking. RESULTS/ANTICIPATED RESULTS: Dynamic control (1) restored blood flow in non-functional tumor vessels and (2) increased and then transiently reversed blood flow in functional vessels. Vessel diameters did not change, suggesting that shunting of systemic blood to the tumor vasculature accounted for the observed changes. Dynamic control augmented tumor responses in our regional therapy model of melanoma. Increases in DNA adduct formation (melphalan mechanism of action) detected by IHC, decreased tumor growth, and increased survival were observed with dynamic control. There was no increased limb toxicity. Similarly, dynamic control augmented responses in our systemic therapy model (decreased tumor growth and improved survival). We anticipate that dynamic control will improve trafficking of effector T cells in the next set of experiments. DISCUSSION/SIGNIFICANCE OF IMPACT: Heterogeneous responses to systemic therapies represent a major gap in current cancer treatment. An essential requirement for any effective therapy is its ability to reach tumor via the tumor-associated vasculature. We have therefore developed an approach to enhance drug delivery (dynamic control), which we also plan to test in clinical trials.

